# Hemicellulosic Biogels: A Fundamentally New Sustainable Platform Approach to Address Societal Grand Challenges

**DOI:** 10.3390/gels11090722

**Published:** 2025-09-10

**Authors:** Ali Ayoub, Lucian Lucia

**Affiliations:** 1 Department of Forest Biomaterials, College of Natural Resources, North Carolina State University, Raleigh, NC 27695, USA; lalucia@ncsu.edu; 2 Department of Chemistry, College of Sciences, North Carolina State University, Raleigh, NC 27695, USA

**Keywords:** hemicellulose, gels, water treatment, coatings, adhesives, food and beverages, agricultural solution, biomedical

## Abstract

The global issues of resource depletion and environmental pollution have led to increased interest in a circular bioeconomy focusing on converting renewable biomass into functional biomaterials. This article explores the transformative potential of hemicellulosic biogels as a sustainable platform to address critical societal challenges, such as water scarcity, food solutions and environmental pollution. Derived from hemicelluloses, an abundant and underutilized polysaccharide in lignocellulose biomass, these biogels offer a fundamentally new approach to developing high-performance, ecofriendly based materials. The review examines their development, characterization, and diverse applications in water treatment, food, agriculture, adhesive and coating systems. In water treatment, these gels exhibit exceptional performance, demonstrating a maximum NaCl uptake of 0.26 g/g and rapid pseudo-second-order adsorption kinetics for desalination. They also show high selectivity for heavy metal removal, with a remarkable binding capacity for lead if 2.9 mg/g at pH 5. For adhesive and coating applications, hemicellulose crosslinked with ammonium zirconium carbonate (AZC) forms water-resistant gels that significantly enhance paper properties, including gloss, smoothness, liquid resistance, and adhesive strength. Furthermore, hemicellulosics exhibit controlled biodegradation in physiological solutions while maintaining their mechanical integrity, underscoring their broad application promise. Overall, this review highlights how hemicellulose-based hydrogels can transform a low-value byproduct from biorefinery into high-performance solutions, contributing significantly to a sustainable economy.

## 1. Introduction

The 21st century is characterized by a rising range of interconnected global challenges such as escalating water scarcity, food security, and unabated environmental pollution [1]. Hemicelluloses are emerging as a particularly promising approach to address, to some degree, these challenges due to their global abundance, renewable origins, and inherent biodegradability [2]. This class of polysaccharides, embedded within the intricate architecture of plant cell walls, offers a unique balance of chemical versatility and functional adaptability. Their name alludes to their resemblance to cellulose, the world’s most abundant biomaterial, yet the abundance of hemicelluloses available offer a veritable cornucopia of feedstocks for the generation of new materials. Their main differences to cellulose are their non-crystalline nature, varieties of functional groups, and ease of processability [3].

Yet, despite their advantageous properties and potential to be isolated from the forest products industry, hemicelluloses remain outstandingly underexploited. These pressing issues necessitate a fundamental shift from staid, defunct, and traditional linear economic models towards a regenerative circular bioeconomy. This paradigm champions the efficient and sustainable utilization of renewable biological resources to produce value-added materials, energy, and services, thereby minimizing waste and maximizing resource efficiency [4]. Within this transformative framework, the development of novel biomaterials derived from abundant and renewable biomass sources is paramount. Bio-based alternatives provide benefits such as renewability, biodegradability, lower carbon footprint, and improved biocompatibility compared to petroleum-based materials [5]. Among the vast array of natural polymers, hemicelluloses stand out as particularly promising candidates due to their ubiquitous availability, diverse structural architectures, and inherent chemical versatility, making them ideal precursors for a new generation of sustainable materials. Hemicelluloses constitute the second most abundant group of polysaccharides in lignocellulosic biomass, typically accounting for 20–35% of the dry weight of plant cell walls. Their global abundance is immense, with approximately 0.41 billion tons available annually in the U.S. alone, primarily as a byproduct of the pulp and paper industry [6,7]. Despite this vast availability, hemicelluloses remain largely underutilized; a significant proportion is currently incinerated for low-value energy recovery, particularly in pulp and paper processes, owing to their comparatively lower heating value than lignin [8]. This practice represents a considerable economic and environmental opportunity cost, underscoring the urgent need for their valorization into higher-value products.

**Figure 1:**
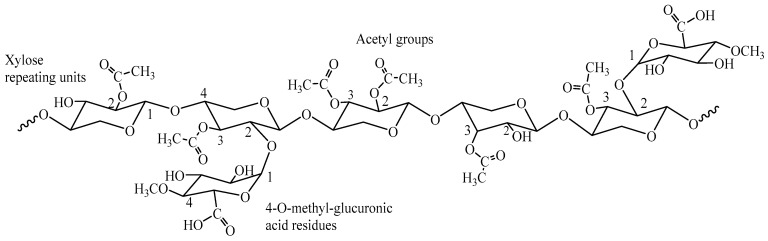
O-acetyl-4-O-methyl-glucoronoxylan: The major hemicellulose in hardwoods.

**Figure 2:**
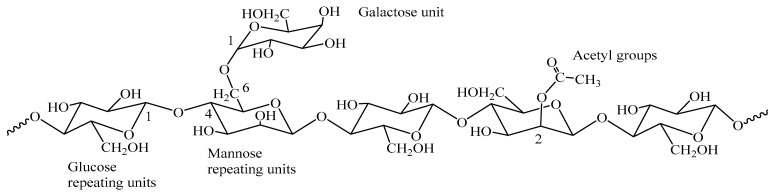
O-acetyl-galactoglucomannan: The major hemicellulose in softwoods.

**Figure 3:**
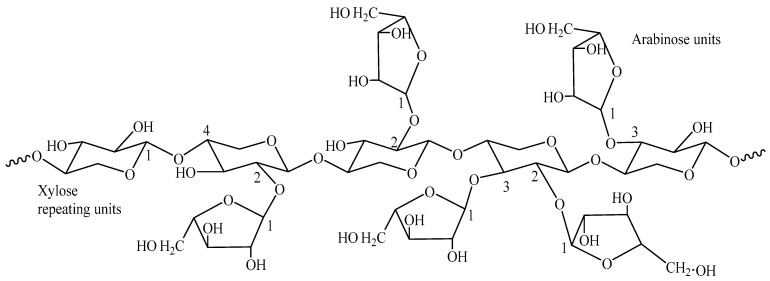
Arabinoxylan: The major hemicellulose in switchgrass.

**Figure 4:**
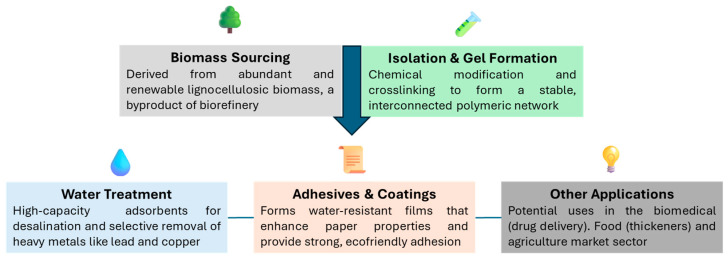
A visual representation of the journey from underutilized byproduct to high-value multifunctional biomaterials.

Hemicelluloses are a heterogeneous class of branched polysaccharides, distinct from cellulose in their amorphous structure, lower molecular weight, and diverse monosaccharide composition. Common examples include O-acetyl-4-O-methyl-glucuronoxylan in hardwoods and arabinoxylan in grasses (Figure 1, Figure 2 and Figure 3). This structural diversity, while presenting complexities in uniform processing, simultaneously offers unique levers for tailored chemical modification and functionalization. The strategic integration of biorefineries plays a pivotal role in transforming these agricultural and industrial byproducts into a diverse portfolio of high-value bioproducts, such as biogels for water treatment, adhesives, and other business markets (Figure 4). This valorization pathway not only enhances economic competitiveness but also significantly contributes to the overarching goals of sustainability [7]. The substantial contribution of the U.S. bioeconomy, valued at $369 billion annually, with biobased chemical production projected to reach 25% of target commodities by 2030 [9], further highlights the critical importance of leveraging hemicelluloses within sustainable biorefinery concepts.

## 2. Biorefinery of Hemicelluloses

A crucial initial step in valorizing hemicelluloses involves their efficient extraction from lignocellulosic biomass. The chosen extraction method primarily utilized sodium hydroxide (NaOH), a chemical already employed and recovered in conventional kraft mills, thereby aligning with existing industrial infrastructure and promoting a more sustainable process. The biomass materials can be processed into fine flour to facilitate subsequent chemical treatments. Here are two methods of hemicellulose extraction [10]:

### 2.1. Extraction of Hemicelluloses Under Alkaline Conditions After Bleaching by NaClO_2_ [10,11,12,13]

**Figure 5:**
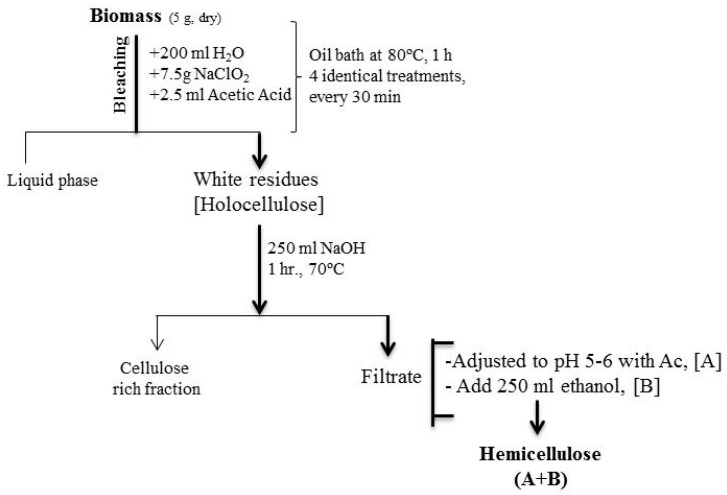
Scheme for the extraction of hemicellulose after bleaching by NaClO_2_.

This method, depicted in Figure 5, commenced with a bleaching step to remove lignin and prepare holocellulose. Five grams of dried biomass were treated with sodium chlorite (NaClO_2_) and glacial acetic acid in an oil bath at 80 °C for one hour, with repeated additions of reagents every 30 min for a total of four identical treatments. This process effectively delignified the biomass, yielding a white holocellulose residue. The holocellulose was then subjected to alkaline extraction using varying concentrations of sodium hydroxide (5%, 10%, and 18% NaOH) at 70 °C for one hour. After filtration to separate α-cellulose, the filtrate, rich in hemicellulose, was neutralized with acetic acid and subsequently precipitated by the addition of isopropanol (IPA). The precipitated hemicellulose was then purified through repeated washing and freeze-drying to obtain a fine powder.

The yields of hemicelluloses obtained by this method are summarized in Table 1. The results indicated that 10% NaOH consistently provided a good measure of hemicellulose content in the original wood, with yields of 28% for pinewood, 35% for coastal Bermuda grass, and 32% for switchgrass.

### 2.2. Direct Alkaline Extraction of Hemicellulose

**Figure 6:**
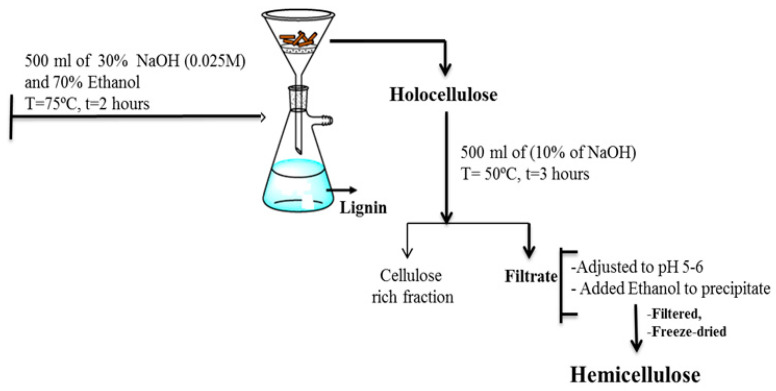
Scheme for the extraction of hemicellulose from biomass (i.e., pine wood, switchgrass, and costal Bermuda grass) [10].

A slightly modified procedure based on Karaaslan et al. (2010) [14] was also employed, as depicted in Figure 6. This cost-effective method involved a two-step alkaline extraction. First, the biomass (5 g) was stirred in 0.025 M NaOH (30%) and 70% ethanol at 75 °C for 2 h to solubilize lignin. The suspension was then filtered to remove lignin. The remaining solids were transferred to a 10% NaOH solution and stirred for 3 h at 50 °C to solubilize hemicelluloses. pH of the filtrate was adjusted to 5.3 with HCl before precipitation of hemicellulose by adding three times its volume of 95% ethanol. The precipitated hemicellulose was then dialyzed and freeze-dried. This method proved effective, yielding 23% hemicellulose from pine wood. Switchgrass and coastal Bermuda grass yielded 26.9% and 29.1%, respectively, also consistent with previous findings. Softwoods like pine, with higher lignin content, typically require initial lignin removal steps for efficient polysaccharide extraction.

### 2.3. Molecular Weight Characterization of Extracted Hemicelluloses

To gain insight into the size and any possible networking of extracted hemicelluloses, their molecular weights were obtained using Gel Permeation Chromatography (GPC). Because hemicellulose is insoluble in tetrahydrofuran (THF), acetylation was necessary. The acetylation involved treating freeze-dried hemicellulose with acetic anhydride in an ionic liquid ([Amim]^+^Cl^−^) at 80 °C. The degree of substitution (DS), which indicates the extent of hydroxyl group acetylation, ranged from 0.03 to 1.25 out of a possible 3.0. Increasing reaction time from 1 to 20 h at 80 °C led to an increase in DS within the range of 0.03 to 1.25 likely approaching 1.25 due to improved diffusion and mobility of reactants, likely due to improved diffusion and mobility of reactants. However, increasing the temperature from 80 °C to 95 °C decreased acetylation, possibly due to degradation of macromolecular hemicellulose into smaller oligosaccharides that were lost during washing [10,13].

The GPC results, as presented in Table 2, summarize the weight-average (M_w_) and number-average (M_n_) molecular weights and polydispersity (PD) for switchgrass hemicellulose extracted at different NaOH concentrations. The 10% NaOH extraction consistently yielded the highest M_w_ (85,700 g/mol), along with a yield of 27%, suggesting severe disruption of the holocellulose macrostructure during this alkaline treatment, leading to the release of larger molecular weight hemicelluloses. In contrast, the 18% NaOH extraction resulted in the lowest molecular weight (3500 g/mol) and a significant yellow discoloration, indicating substantial degradation. Based on both yield and molecular weight, the 10% NaOH extraction method was deemed optimal. Similar trends were observed in other studies, where higher NaOH concentrations led to lower molecular weight hemicelluloses due to degradation. The study also reported, for the first time, the estimated molecular weights of hemicellulose isolated from lignified switchgrass under alkaline conditions using this novel method. Overall, the weight-average molecular weights (M_w_) for pine wood, switchgrass, and coastal Bermuda grass hemicelluloses (extracted with 10% NaOH) were 79,800, 85,710, and 83,200 g/mol, respectively (Table 3) [10,12].

## 3. Hemicellulose-Based Gels: Fundamental Properties and Advanced Processing

Among the various high-value products derived from hemicelluloses, gels have emerged as a particularly promising class of biomaterials. Hemicellulose-based gels are three-dimensional, hydrophilic polymeric networks capable of absorbing and retaining substantial quantities of water or aqueous solutions (often hundreds to thousands of times their dry weight) without dissolving [2,15]. Their inherent attributes, namely, renewability, biodegradability, biocompatibility, and non-toxicity, make them exceptionally well-suited for applications demanding environmentally benign and physiologically compatible materials. However, the native properties of hemicelluloses, such as their inherent hydrophilicity and relatively low mechanical strength, often necessitate chemical modification to expand their functional capabilities and broaden their application scope.

Common strategies involve the incorporation of anionic groups (e.g., carboxyl), cationic groups, or hydrophobic segments, which can significantly alter their swelling behavior, mechanical properties, and interaction with various substances [16]. Furthermore, the scalability and economic viability of producing hemicellulose hydrogels for industrial applications hinge on the adoption of advanced processing techniques. Reactive extrusion, a continuous, solvent-free, and highly efficient thermo-mechanical process, has proven particularly effective in transforming hemicelluloses into high-value, stimuli-responsive hydrogels. This technique enables in situ chemical reactions like crosslinking and shapes the material at the same time, improving functionality and scalability in an environmentally friendly way [17,18].

### Principles of Gel Formation and Swelling Dynamics

The formation of hemicellulose-based hydrogels involves a gelation process, leading to the establishment of a stable, interconnected polymeric network. Various mechanisms can induce this network formation, including physical crosslinking through non-covalent interactions such as precipitation by altering solvent conditions [19], freeze–thaw cycles which promote polymer chain aggregation and physical entanglement [20], temperature-induced chain coalescence leading to the formation of stable junction zones, and self-segregation of hydrophobic/hydrophilic segments in amphiphilic hemicellulose derivatives, resulting in micellar or network formation [21].

**Figure 7:**
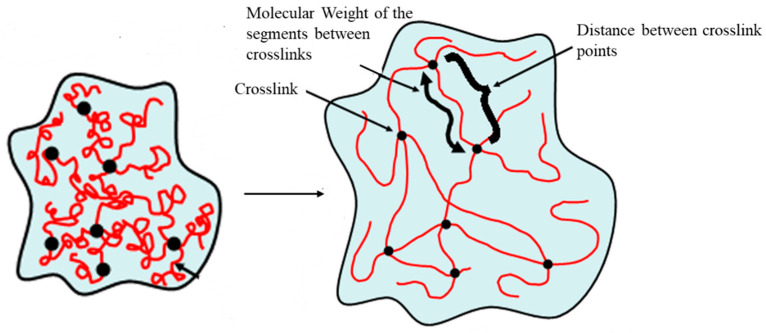
Representation of a hydrogel in its somewhat condensed (**left**) and highly swollen (**right**) states. Solid dots indicate crosslinked points of the biogel chains.

Additionally, chemical crosslinking involves the formation of stable covalent bonds between polymer chains, imparting enhanced mechanical stability and durability, with common crosslinking agents including diethylenetriaminepentaacetic acid (DTPA), citric acid (CA), or ammonium zirconium carbonate (AZC) (Figure 7) [11,22]. Reactive extrusion is particularly advantageous for facilitating these chemical crosslinking reactions in a continuous and efficient manner, especially for applications like controlled drug delivery [12]. Furthermore, polyelectrolyte complexes (PECs) are formed through electrostatic interactions between oppositely charged polyelectrolytes, such as anionic DTPA-modified hemicellulose and cationic chitosan, which can significantly enhance the hydrogel’s ability to absorb saline solutions due to their unique charge-driven network formation [13].

**Figure 8:**
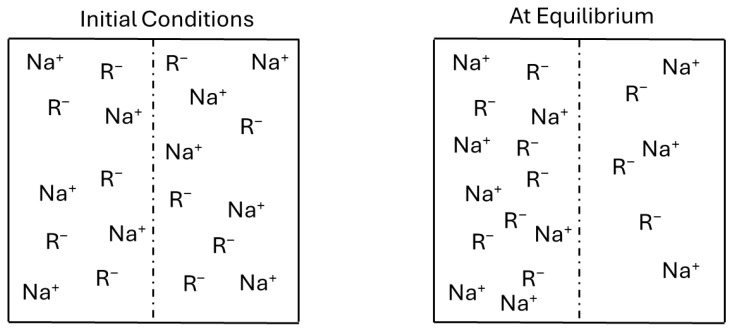
The partitioning of ionic species between two solutions is separated by a semi-permeable membrane. The membrane is permeable to the mobile ions (Na^+^ and Cl^−^) and impermeable to the non-diffusible polyanions (R^−^). The presence of the fixed negative charges on the biogel’s polymer chains (R^−^) creates an unequal distribution of ions across the membrane. To balance the charge, a higher concentration of mobile counter-ions (Na^+^) accumulates inside the gel. This high concentration of ions inside the gel creates significant osmotic pressure, drawing water molecules into the network. This osmotic force is the primary driving force behind the swelling of the hydrogel, leading to its highly expanded state at equilibrium.

The swelling behavior of hemicellulose-based hydrogels, a critical determinant of their performance across diverse applications, is profoundly influenced by the physico-chemical properties of the surrounding fluid and the intrinsic characteristics of the polymer network. This behavior is governed by a complex interplay of thermodynamic forces, primarily osmotic pressure, entropy of mixing, and hydrogen bonding, all operating within the framework of Donnan’s equilibrium [23]. Donnan’s equilibrium describes the distribution of ions across a semi-permeable membrane (the hydrogel network) when a fixed charge is present within the gel (Figure 8).

This charge imbalance generates osmotic pressure that drives water into the network. The entropy of mixing, described by the change in Gibbs free energy (ΔG = ΔH − TΔS), also contributes to swelling; as water enters the network, the polymer chains gain conformational freedom, leading to an increase in entropy (ΔS), which favors swelling [24]. Furthermore, the abundance of hydroxyl and carboxyl groups on hemicellulose chains facilitates extensive hydrogen bonding with water molecules, enhancing hydration and ensuring that a significant proportion of the absorbed water is tightly bound within the hydrogel matrix [25]. Key environmental factors influencing hydrogel absorbency include salinity, where in the presence of salts (e.g., 0.9% NaCl), the absorption capacity is typically reduced due to the electrostatic screening of anionic groups by counter-ions, although PEC hydrogels can exhibit remarkable saline uptake due to their distinct charge-driven swelling mechanisms [26].

pH also plays a crucial role, with swelling generally increasing as pH approaches neutrality due to the deprotonation of carboxyl groups within the hemicellulose backbone, leading to enhanced electrostatic repulsion and higher osmotic pressure [27], while at low pH, protonation can lead to hydrogen bonding and reduced absorbency [28]. The type and valency of ionic species in the solution significantly impact swelling, as multivalent cations (e.g., Ca^2+^) form stronger ionic crosslinks or chelates, leading to a more pronounced reduction in swelling compared to monovalent ions (e.g., Na^+^) [29]. Finally, while temperature typically causes a decrease in swelling due to increased kinetic energy of water molecules, some specific thermo-responsive systems may exhibit the opposite behavior [29], and external pressure (e.g., 300 kPa) can also significantly compress the hydrogel network, thereby reducing its swelling capacity These intricate physicochemical properties are critical considerations for optimizing hemicellulose gels for specific applications, particularly in water treatment

## 4. Hemicellulose-Based Hydrogels in Water Treatment

The global water crisis stands as one of the most significant challenges of our time, profoundly impacting human health, economic development, and ecological stability. Driven by rapid population growth, industrial expansion, and the pervasive effects of climate change, the demand for clean, potable water far outstrips the available supply in many regions [30]. Concurrently, the increasing discharge of toxic ionic species, particularly heavy metals, from industrial and agricultural activities into natural water bodies poses a severe threat to aquatic ecosystems and human well-being.

Traditional water treatment methodologies, such as chemical precipitation, oxidation/reduction, and electrochemical treatments, while effective to varying degrees, often present significant drawbacks. Chemical precipitation, for instance, can be inefficient and generates substantial volumes of sludge, necessitating further disposal. Chemical reduction/oxidation processes demand large quantities of chemical reagents, leading to increased operational costs and potential secondary pollution. Electrochemical treatments, though advanced, frequently incur high energy consumption and operational expenses [31]. Similarly, while activated carbon has long been a popular adsorbent for heavy metal removal, its high cost often limits its economic viability for large-scale industrial applications [32]. These limitations collectively highlight the pressing need for alternative, low-cost, and high-performance solutions for water desalination and heavy metal remediation.

The search for such alternatives has intensified the focus on renewable biomaterials that can offer performance properties comparable to, or even superior to, petroleum-based synthetic materials. The inherent advantages of biomaterials, including a more stable raw material supply, reduced cost fluctuations, improved biocompatibility, and the utilization of renewable resources, position them as highly attractive candidates. Among these, hemicellulose emerges as a material with exceptional potential for water treatment applications. Its chemical modification offers a versatile pathway to engineer materials with unique properties, thereby increasing the value and utility of this abundant biopolymer [33]. One particularly promising application area is the development of absorbent gels. These materials have garnered significant attention due to their remarkable liquid uptake capabilities, their ability to swell reversibly without disintegration in response to external stimuli, and their inherent biocompatibility. The mechanical stability of these hydrogels during swelling is attributed to the presence of chemical and/or physical cross-links introduced between their macromolecular chains, providing both flexibility and sufficient strength.

Ayoub et al. [22] developed high-value gel products specifically engineered for water desalination and heavy metal remediation, leveraging the abundant and renewable nature of hemicellulose (HC) as a key building block. This innovative approach combined HC with chitosan (CS) and diethylenetriaminepentaacetic acid (DTPA) to create robust, biocompatible materials with enhanced absorption and mechanical properties. Conducted at a laboratory scale, the study focused on synthesizing and characterizing hemicellulose-based formulations, which were then transformed into foam structures via freeze-drying. The resulting gels demonstrated significant potential for environmental applications, such as efficiently removing salts and heavy metals from water, highlighting the broader impact of valorizing underutilized biomass like hemicellulose into sustainable, high-performance products, a concept that has inspired real-world commercialization efforts, including by the Raleigh-based startup *Tethys* Incorporation, which specializes in plant-based superabsorbent biopolymers derived from similar carbohydrate sources for eco-friendly absorbent technologies.

**Figure 9:**
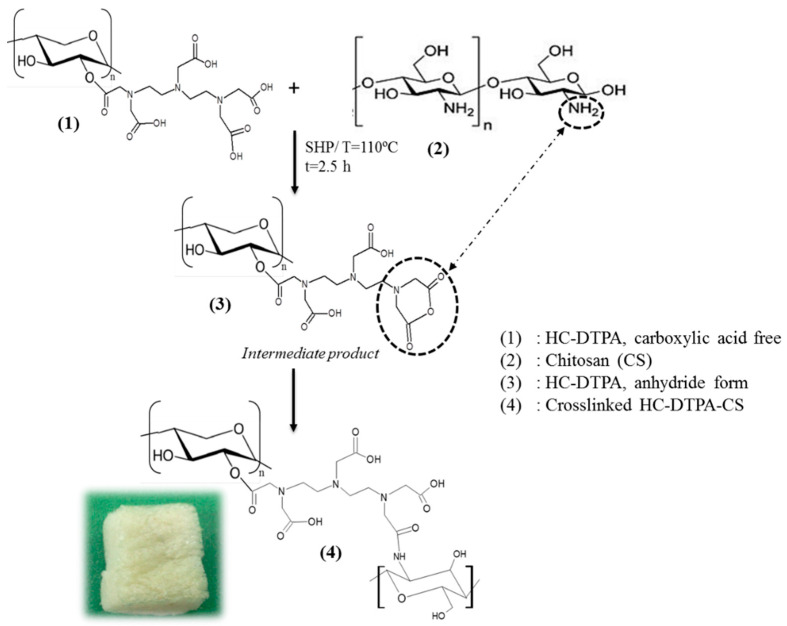
Simplified schema of the reaction between hemicellulose-DTPA and chitosan.

To achieve this, the DTPA-modified hemicellulose was crosslinked with chitosan, another renewable biopolymer renowned for its biocompatibility, biodegradability, and low toxicity. While chitosan-based hydrogels often suffer from limited mechanical stability without reinforcement, this crosslinking strategy formed a strong, covalent network between the two polymers, improving overall durability and functionality. The reaction involved mixing a chitosan solution (dissolved in water and glacial acetic acid) with a 1% hemicellulose-DTPA solution, stirring at 110 °C for 2.5 h under controlled water evaporation with a condenser. After cooling, the mixture was freeze-dried to yield the hemicellulose-DTPA-chitosan aerogel. A simplified schematic of this process, illustrating the key interactions between the carboxylic groups of DTPA-modified hemicellulose and the amine groups of chitosan, is shown in Figure 9.

The HC-DTPA-CS gel exhibited substantially higher water and saline absorption capacities and remarkably lower weight loss when exposed to water and saline solutions (Table 4). Notably, a *negative* weight loss was observed for HC-DTPA-CS after exposure to saline solution, indicating a strong adsorption of salt from the solution, rather than simply absorbing water. This enhanced performance was consistent across hemicelluloses extracted from different biomass sources (SG, PW, CBG). In contrast, commercial cellulose foam showed significantly lower absorption and higher weight loss.

FTIR analysis provided crucial evidence for successful crosslinking. As shown in Figure 9, the FTIR spectrum of the HC-DTPA-CS foam revealed a new peak at 1716 cm^−1^, which is characteristic of the stretching band of carbonyl groups in an amide bond. This finding strongly supports the notion that hemicellulose-DTPA is covalently linked to chitosan via reactions between the amine groups of chitosan and the carboxylic groups of DTPA-modified hemicellulose, creating an amide-based crosslink.

The reaction conditions significantly influenced the foam properties. The effect of reaction time (0.5 to 2.5 h) on the properties of switchgrass hemicellulose-DTPA/chitosan foam was investigated, keeping the ratio, pH, and temperature constant (Table 4). Both water and saline absorption increased with increasing reaction time, while weight loss decreased, signifying a stronger crosslinked structure. It was also observed that reactions conducted at 90 °C did not produce stable foams, as shown by the products displaying very low strength and collapsing upon immersion in water, highlighting the importance of optimal reaction temperature.

Furthermore, the ratio of hemicellulose-DTPA to chitosan was a critical factor. The concentration of hemicellulose-DTPA varied while keeping chitosan fixed. Titration revealed that SG-hemicellulose grafted with DTPA had approximately 835 mequivalents per 100 g of material, while chitosan had 525 mequivalents of amine per 100 g. Increasing the hemicellulose-DTPA to chitosan ratio led to an increase in both water and saline absorption. This correlation is consistent with the increased content of hydrophilic carboxylic acid groups from DTPA, which enhances the water-holding capacity of the hydrogel.

This detailed synthesis and characterization of hemicellulose-DTPA-chitosan foams laid the groundwork for evaluating their performance in the specific applications of water desalination and heavy metal removal, which will be discussed in subsequent sections.

Beyond desalination, the ability of the hemicellulose-DTPA-chitosan gels to remove toxic heavy metal ions from wastewater streams was a critical area of investigation. The adsorption of nickel (II) (Ni^2+^), copper (II) (Cu^2+^), and lead (II) (Pb^2+^) ions was studied at different pH levels (4 and 5) using switchgrass-based HC-DTPA-CS foam, with initial metal ion concentrations of both 5000 PPB (parts per billion) and 100 PPB. pH of the solutions was adjusted using 0.1 N HCl and 0.1 N NaOH solutions.

The results demonstrated the significant heavy metal uptake capacity of the developed biosorbent and revealed interesting selectivity patterns:

**Copper (II) (Cu^2+^) Uptake:** The maximum uptake of Cu(II) ions was observed at pH 4, with sorption capacities of 1.17 mg/g at an initial loading of 5000 PPB and 0.07 mg/g at 100 PPB. At pH 5, the uptake slightly decreased to 0.95 mg/g (5000 PPB) and remained at 0.07 mg/g (100 PPB).

**Nickel (II) (Ni^2+^) Uptake:** For Ni(II) ions, the maximum uptake occurred at pH 5, reaching 1.37 mg/g at 5000 PPB and 0.14 mg/g at 100 PPB. At pH 4, the uptake was slightly lower at 1.20 mg/g (5000 PPB) and 0.10 mg/g (100 PPB).

**Lead (II) (Pb^2+^) Uptake:** The foam material exhibited a remarkably high selectivity for Pb^2+^ ions. The maximum binding capacity for Pb^2+^ was 2.9 mg/g at pH 5 (for an initial concentration of 5000 PPB), and 0.18 mg/g at 100 PPB. This indicates a strong affinity of the biosorbent for lead ions compared to copper and nickel. At pH 4, Pb^2+^ uptake was 2.40 mg/g (5000 PPB) and 0.20 mg/g (100 PPB), still demonstrating superior performance.

The observed pH dependence of heavy metal uptake is consistent with the principles of adsorption on polyelectrolyte materials. At lower pH, the concentration of hydrogen (H^+^) ions is higher, leading to increased competition between H^+^ ions and metal ions for the available binding sites on the foam. This competition reduces the overall metal ion uptake. As pH increases towards the isoelectric point and beyond, the functional groups on the adsorbent surface, such as the carboxyl groups from DTPA and amine groups from chitosan, become deprotonated. This results in a more negative charge on the surface, which promotes stronger electrostatic attractions between the positively charged metal ions (Pb^2+^, Cu^2+^, Ni^2+^) and the negatively charged binding sites. The high selectivity for Pb^2+^ can be attributed to the specific chelating properties of the DTPA functional groups, which form very strong complexes with lead ions, a characteristic well-documented for polyamino carboxylic acids. This strong chelation allows the foam to effectively sequester lead even in the presence of other metal ions.

The ability of these hemicellulose-DTPA-chitosan foams to effectively remove various heavy metals, particularly their high selectivity for lead, positions them as highly promising and sustainable biosorbents for industrial wastewater treatment and environmental remediation efforts.

However, significant research gaps remain that limit the broader adoption of hemicellulose-based hydrogels. First, the scalability of the synthesis process is underexplored; laboratory-scale freeze-drying and high-temperature (110 °C) crosslinking may not be energy-efficient or cost-effective for industrial-scale production, necessitating studies on alternative, low-energy processing methods like ambient-temperature crosslinking or spray-drying. Second, the long-term stability and recyclability of these hydrogels in real-world wastewater streams—often containing complex mixtures of organic pollutants, salts, and competing ions—are not well-characterized. For example, while the foams show high selectivity for Pb^2+^, their performance in multi-metal or high-salinity environments remains unclear, potentially limiting their efficacy in industrial settings. Third, the environmental fate of these hydrogels, including their biodegradability and potential release of microplastics or residual DTPA, requires further investigation to ensure they do not introduce secondary pollutants. Additionally, comparative studies with other bio-based hydrogels (e.g., starch or pectin-based) are sparse, leaving gaps in understanding hemicellulose’s competitive edge in mechanical strength or adsorption kinetics. Addressing these gaps through pilot-scale testing, lifecycle assessments, and kinetic modeling could bridge the divide between laboratory success and industrial viability, positioning hemicellulose-based hydrogels as a cornerstone of sustainable water treatment technologies.

## 5. Hemicellulose-Based Gels in Adhesive and Coating Systems

The global industrial landscape is increasingly seeking sustainable alternatives to conventional petroleum-derived adhesives and coatings, driven by environmental concerns, regulatory pressures, and the desire for enhanced performance characteristics. Traditional synthetic adhesives often pose challenges related to their non-renewable origins, volatile organic compound (VOC) emissions, and limited biodegradability [34]. This growing demand for eco-friendly, high-performance binding agents and surface treatments has brought biopolymers to the forefront of material science research. Among these, hemicellulose-based gels offer a particularly compelling solution, leveraging their inherent renewability, biodegradability, and versatile chemical structures to develop advanced adhesive and coating systems. Their ability to form robust, water-resistant films and provide strong adhesion to various substrates positions them as promising candidates for a wide array of industrial applications, including paper and packaging, wood products, and specialty coatings.

The development of effective hemicellulose-based adhesives and coatings hinges on overcoming certain inherent limitations of native hemicelluloses, such as their high hydrophilicity and relatively low mechanical strength in wet environments. To address these challenges and enhance their performance, chemical modification and crosslinking strategies are typically employed [34]. A key approach involves the use of crosslinking agents that can react with the hydroxyl groups abundant in hemicellulose, forming stable three-dimensional networks that improve water resistance, mechanical integrity, and adhesive properties. One highly effective crosslinking agent explored for this purpose is ammonium zirconium carbonate (AZC) [35].

**Figure 10:**
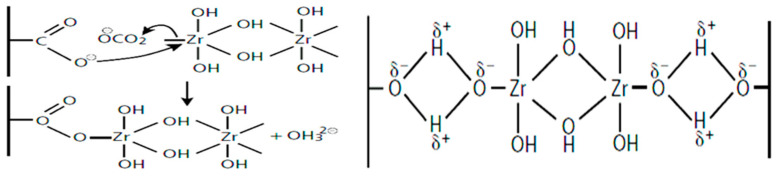
Interaction between zirconium and the carboxylic and free hydroxyl groups of hemicellulose.

AZC functions as a non-toxic, inorganic crosslinker that reacts with various functional groups, including hydroxyls and carboxyls, to form stable covalent bonds (Figure 10). When hemicellulose is crosslinked with AZC, it forms water-resistant gels that can be effectively utilized as coatings or adhesives. The selection of AZC is advantageous due to its environmentally benign nature and its ability to impart significant improvements in material properties, particularly in terms of water resistance and mechanical robustness, which are critical for adhesive and coating applications [36]. The development process typically involves preparing a solution or dispersion of hemicellulose, followed by the addition of the AZC crosslinker. The mixture is then applied to the desired substrate (e.g., paper, wood) and cured, often with heat, to facilitate the crosslinking reaction and form a stable, insoluble film. Precise control over the concentration of hemicellulose, the amount of AZC, and the curing conditions is crucial for optimizing the final properties of the adhesive or coating, ensuring adequate film formation, mechanical strength, and adhesion.

### 5.1. Performance Characteristics in Paper and Packaging Applications

Hemicellulose-based hydrogels, particularly those crosslinked with ammonium zirconium carbonate (AZC), show significant promise as functional additives and coatings in the paper and packaging industry. This sector continually seeks sustainable materials to enhance product performance, minimize environmental impact, and improve process efficiency. Applying hemicellulose-AZC gels as paper coatings transforms surface characteristics and elevates the overall quality of paper products by forming water-resistant gels that deliver multiple benefits.

These gels enhance paper gloss, with research demonstrating improvements of up to 30.6% [37]. The formation of a smooth, uniform film on the paper surface increases light reflection, resulting in a more aesthetically appealing appearance ideal for printing, packaging, and specialty paper products. Additionally, the coatings improve smoothness by reducing surface roughness to as low as 7.5 µm [37]. This finer, more even texture enhances print quality, enabling sharper images and text, while also providing a pleasant tactile feel (Table 5).

The gels also significantly boost liquid resistance, a critical property for paper and packaging materials. Hemicellulose-AZC coatings create a water-resistant barrier, achieving an impressive Hydrostatic Pressure Test (HST) value of 0.7 hr [37]. This high resistance to moisture prevents absorption, ensuring product integrity in packaging applications. The crosslinked AZC network forms a physical barrier and reduces the hydrophilic nature of hemicellulose, enhancing water repellency.

Furthermore, these hemicellulose-based materials exhibit strong adhesive properties, with adhesive strengths reaching up to 3.46 MPa [38]. This robustness supports various bonding applications, such as laminating, carton sealing, and label adhesion, within the paper and packaging industry. The strong adhesion results from multiple hydrogen bonds and covalent linkages between the hemicellulose-AZC network and the cellulose fibers in the paper substrate.

The mechanisms underlying these enhanced properties involve the formation of a stable, crosslinked network on the paper surface. Upon curing, AZC decomposes, releasing ammonia and carbon dioxide, enabling zirconium ions to form coordinate covalent bonds (chelates) with oxygen atoms from hydroxyl and carboxyl groups on hemicellulose chains, bridging multiple polymers while competing with AZC self-crosslinking. This creates a dense and water-resistant film that not only physically fills the pores and irregularities on the paper surface, leading to increased smoothness and gloss, but also reduces the penetration of water due to its crosslinked, less hydrophilic nature. The strong interfacial adhesion is a result of both chemical interactions (covalent bonds, hydrogen bonding) and physical interlocking between the hydrogel network and the paper fibers.

### 5.2. Hemicellulose Versus Lignin-Based Adhesives in Bio-Based Formulations

Hemicellulose and lignin, both derived from lignocellulosic biomass, serve as promising sustainable alternatives to synthetic adhesives, yet they exhibit distinct chemical and functional properties that influence their adhesive performance [39,40,41,42]. Hemicellulose, a brancher polysaccharide rich in sugars like xylose and mannose, offers advantages in biodegradability, lower cost due to its abundance as a pulping by product, and tunable viscosity for easier processing, but it often requires modifications such as crosslinking with agents like chitosan or poly(vinyl amine) to enhance water resistance and bond strength, making it suitable for dry indoor applications like wood composites [43]. In contrast, lignin, an aromatic polymer with phenolic structures, inherently provides superior hydrophobicity, rigidity, and microbial resistance, acting as a natural binder in plant cell walls and enabling stronger wet shear strength in formulations like lignin-phenol-formaldehyde or lignin-glyoxal resins, though it demands energy-intensive modifications (e.g., demethylation) to improve reactivity and can result in higher viscosity and darker coloration under stringent curing conditions [44,45]. Recent advances highlight hybrid systems combining both, where lignin’s crosslinking enhances hemicellulose’s structural reinforcement, achieving plywood strengths up to 3.2 MPa dry and 1.73 MPa wet, positioning them as competitive, formaldehyde-free options in wood panels and beyond [46]. While lignin excels in high-humidity environments, hemicellulose’s versatility in blends underscores its role in eco-friendly innovations, with ongoing research focusing on valorizing uncondensed lignins and hemicellulose-rich sidestreams for fully bio-based adhesives [47,48].

Building on the core differences (Table 6), recent developments emphasize lignin’s potential in advanced urea-formaldehyde (UF) adhesives, where modifications improve bonding performance and sustainability, reducing reliance on fossil-based components [49]. For instance, lignin-based polymers are increasingly used in wood panels for their eco-friendly profile, with tannin and soy integrations further enhancing adhesion while minimizing emissions. Hemicellulose, meanwhile, shines in hydrogel composites and self-adhesive materials, leveraging its hydrogen-bonding capabilities for stretchable, conductive applications beyond traditional wood bonding, such as in food packaging and biomedical fields [48]. Compared to other polysaccharides like starch, chitosan, or pectin, hemicellulose offers superior compatibility with cellulose in wood matrices but shares challenges in water sensitivity, often addressed through molecular weight adjustments or blends. Higher molecular weight variants reduce viscosity while boosting strength, outperforming starch in non-gelatinized forms but lagging behind chitosan’s innate crosslinking. Emerging trends include hemicellulose extraction advancements for hydrogel engineering and lignin valorization in composites, with bioresidues providing cost-effective sources; for example, organosolv lignin variants are more hydrophobic and suitable for high-value adhesives, while hemicelluloses from plant walls contribute to biomechanical enhancements in energy storage and construction materials. These innovations align with broader sustainability goals, demonstrating competitive performance against traditional adhesives through catalytic depolymerization and multifunctional formulations [50,51]

### 5.3. Advantages and Future Directions in Adhesive and Coating Applications

Hemicellulose-based hydrogels, particularly those crosslinked with ammonium zirconium carbonate (AZC), offer distinct advantages over traditional synthetic adhesives and coatings. Derived from renewable lignocellulosic biomass, these hydrogels promote sustainability by reducing dependence on fossil resources and supporting a circular bioeconomy. Their biodegradability minimizes environmental impact at the end of their lifecycle, addressing concerns related to plastic waste. Additionally, hemicelluloses and AZC are generally non-toxic, ensuring safety in manufacturing and end-use applications, such as food packaging. The ability to chemically modify hemicelluloses and adjust crosslinking density enables customization of gel properties, including water resistance, flexibility, and adhesive strength, to meet specific application needs.

Despite these benefits and promising laboratory results, further research is necessary to achieve widespread commercialization. Key areas for development include scaling production through methods like reactive extrusion or other continuous processes to ensure cost-effectiveness and high throughput. Investigating long-term stability, durability, and aging under diverse environmental conditions, such as humidity, temperature fluctuations, and UV exposure, is critical to confirm suitability for various applications. Expanding adhesive performance studies to include substrates beyond paper, such as plastics, metals, and composites, will broaden market potential. Optimizing formulation and processing parameters is essential to reducing production costs and enhancing economic competitiveness. Additionally, incorporating functionalities like antimicrobial properties, flame retardancy, or gas barrier capabilities (e.g., against oxygen) can lead to advanced multi-functional coatings.

## 6. Applications in Biomedical, Food, and Agriculture

While hemicellulosic biogels show significant promise in water treatment and coating systems, their versatile properties, particularly their biocompatibility and biodegradability, open up a broader range of applications in other critical sectors. Expanding the scope of this review to include biomedical, food, and agricultural uses provides a more complete understanding of their potential as sustainable biomaterials.

### 6.1. Biomedical Applications

Hemicellulose-based gels are highly suitable for biomedical applications due to their inherent biocompatibility, non-toxicity, and ability to biodegrade in physiological solutions [52]. Their unique gel structure and chemical versatility allow them to be engineered for specialized functions. For example, they can be utilized as platforms for controlled drug delivery, where the gel matrix can encapsulate and release therapeutic agents over a specific period. In tissue engineering, these materials can be processed into scaffolds that mimic the natural extracellular matrix, providing a supportive environment for cell growth and regeneration. Their high liquid absorption capacity also makes them effective as advanced wound dressings, capable of managing exudate while providing a moist, protective environment that supports the healing process.

### 6.2. Food and Beverage Industry

The utilization of hemicelluloses in the food and beverage industry represents a key pathway for valorizing agricultural byproducts into high-value products [7]. Hemicellulose-based materials can function as natural food additives, replacing synthetic ingredients. Their ability to form stable gels and films makes them ideal as gelling agents, thickeners, and stabilizers, improving the texture, consistency, and shelf life of various food products. This approach aligns with a growing consumer demand for clean-label products derived from renewable resources and contributes to a more sustainable global value chain within the food technology sector.

### 6.3. Agriculture

In agriculture, hemicellulose-based gels offer innovative, eco-friendly solutions for water and nutrient management [53]. Their remarkable liquid uptake capabilities can be leveraged to create superabsorbent biopolymers that enhance soil water retention, reducing the need for frequent irrigation and mitigating water scarcity challenges. By acting as a water reservoir in the soil, they ensure moisture is available to plants over an extended period. Additionally, these gels can be designed to encapsulate fertilizers and pesticides, enabling the controlled release of these compounds and improving their efficiency. This targeted delivery reduces chemical runoffs, minimizes environmental pollution, and optimizes nutrient uptake by crops, contributing to a more sustainable and resource-efficient agricultural system

## 7. Quantitative Benchmarking Against Conventional Materials

To provide a comprehensive understanding of the competitive advantages of hemicellulose-based gels, it is essential to quantitatively benchmark their performance against conventional materials currently used in industry. The data presented in this section highlights the superior or comparable performance of hemicellulose-based gels, demonstrating their viability as a sustainable alternative.

In water treatment, the high selectivity and adsorption capacity of hemicellulose-based gels for heavy metals are a key advantage. While activated carbon (AC) is a widely used adsorbent, its performance varies significantly depending on the source material and activation process. The Pb^2+^ uptake of activated carbons from various sources can range from 1 to over 200 mg/g, with some commercial ACs showing capacities around 40–50 mg/g. However, the hemicellulose-DTPA-chitosan gel, with its Pb^2+^ uptake of 2.9 mg/g at a low initial concentration, shows a high binding affinity at concentrations relevant to real-world industrial effluent. This is particularly notable when considering the high cost and environmental drawbacks associated with the production and regeneration of activated carbon.

For adhesive and coating applications, hemicellulose-based gels offer a compelling alternative to synthetic resins like urea-formaldehyde (UF), which are known for their high formaldehyde emissions. While UF resins provide excellent dry bonding strength (often >1.0 MPa), their wet shear strength is significantly lower. In contrast, the hemicellulose-AZC system demonstrated an impressive adhesive strength of up to 3.46 MPa, highlighting its robust performance. Similarly, the liquid resistance of paper coated with hemicellulose-AZC, measured by a Hydrostatic Pressure Test (HST) value of 2810 s, is competitive with some synthetic and starch-based coatings, showcasing its effectiveness in creating a strong moisture barrier without using petroleum-derived polymers.

Table 7 summarizes the quantitative comparison, demonstrating that hemicellulose-based gels are not merely a “green” alternative but a high-performance solution capable of competing with established materials on a technical level.

## 8. Sustainability, Limitations, and Future Perspectives

The development and application of hemicellulose-based gels are fundamentally rooted in the principles of sustainability, offering a viable pathway toward a circular bioeconomy. The core sustainable advantage of these materials lies in their origin: they are derived from hemicelluloses, which are the second most abundant polysaccharide in lignocellulosic biomass and an underutilized byproduct of the pulp and paper industry. This approach promotes sustainability by reducing the dependence on fossil resources and transforming low-value waste streams into high-performance solutions. Furthermore, the inherent biodegradability of hemicellulose biogels minimizes their environmental impact at the end of their lifecycle, directly addressing global concerns related to plastic waste and pollution. Comprehensive life cycle assessments (LCA) are essential to confirm these environmental benefits, ensuring that the full ecological footprint, from production to disposal, aligns with the goals of sustainability and that their biodegradability does not lead to unintended ecological impacts. Their non-toxic nature, particularly when crosslinked with environmentally benign agents like ammonium zirconium carbonate (AZC), ensures safety in manufacturing and end-use applications, including food contact.

Despite this significant promise, the widespread commercialization of hemicellulose-based gels faces several key limitations. Scalability remains a critical challenge, as energy-intensive and cost-prohibitive laboratory-scale processes like freeze-drying or high-temperature crosslinking are not feasible for industrial applications. Additionally, the long-term performance and durability of these gels in complex, real-world conditions—such as multi-metal wastewater streams or high-humidity adhesive applications—are underexplored. There is also a lack of comparative studies with other bio-based materials, such as starch or pectin hydrogels, which makes it difficult to fully assess hemicellulose’s competitive advantages in cost, strength, or adsorption efficiency. Looking forward, future research should prioritize energy-efficient production methods like reactive extrusion or ambient-temperature crosslinking. Enhancing multifunctionality through novel chemical modifications or hybrid formulations could also improve mechanical and adsorption properties. By addressing these gaps and integrating these materials into biorefinery frameworks, hemicellulose-based gels can transition from promising laboratory innovations to scalable, high-performance solutions, driving a sustainable and resource-efficient future for global environmental and industrial demands.

## 9. Conclusions

Hemicellulose-based gels represent a transformative new platform for developing sustainable materials, leveraging abundant and underutilized lignocellulosic byproducts to address pressing global issues in water treatment, adhesives, and coatings, as well as in other key sectors like food, biomedical, and agriculture. By championing a circular bioeconomy, these biogels offer renewable, biodegradable, and biocompatible alternatives to conventional petroleum-derived materials. Their valorization into high-performance solutions demonstrates a shift from linear economic models toward a regenerative and resource-efficient future.

In water treatment, hemicellulose-based gels show exceptional performance as sustainable adsorbents. Specifically, hemicellulose-DTPA-chitosan crosslinked gels demonstrate a remarkable capacity for water desalination, with a maximum NaCl uptake of 0.26 g/g and rapid pseudo-second-order adsorption kinetics. Furthermore, they exhibit a high selectivity for heavy metal removal, achieving a significant lead (Pb^2+^) uptake of 2.9 mg/g at pH 5. These properties position them as effective and eco-friendly alternatives to traditional, often costly, methods like activated carbon and chemical precipitation.

Similarly, in adhesive and coating systems, hemicellulose-based hydrogels provide a compelling, sustainable solution. When crosslinked with ammonium zirconium carbonate (AZC), these gels form robust, water-resistant films. This crosslinking significantly enhances the performance of paper substrates, improving properties such as gloss, smoothness, and liquid resistance, with an impressive Hydrostatic Pressure Test (HST) value of 2810 s. Their strong adhesive strength, reaching up to 3.46 MPa, makes them suitable for various bonding applications and reduces reliance on volatile organic compound (VOC) emitting synthetic adhesives.

Despite these promising results, key challenges remain for widespread commercialization. Scalability is a critical hurdle, as many current processes are energy-intensive and not yet viable for industrial-scale production. Future efforts must focus on validating long-term performance and durability in complex, real-world environments and expanding their multifunctionality. By addressing these limitations and effectively integrating these materials into established biorefinery frameworks, hemicellulose-based gels can successfully transition from promising laboratory innovations to a cornerstone of a sustainable and resource-efficient global bioeconomy.

## Figures and Tables

**Table 1:** Percentage yield of hemicellulose (HC) obtained by alkali extraction of different kinds of holocellulose from PW, CBG, and SG at different concentrations of NaOH (5, 10, and 18%).

% NaOH	% HC from PW	% HC from CBG	% HC from SG
5	12 ± 0.71	16 ± 0.35	9 ± 0.31
10	28 ± 1.38	35 ± 0.94	32 ± 1.63
18	23 ± 2.10	27 ± 1.97	25 ± 1.15

**Table 2:** The weight-average (*M_w_*), number-average (*M_n_*) molecular weights in g/mol, and the polydispersity (*M_w_*/*M_n_*) of the hemicellulosic fractions isolated from switchgrass, with a comparison to the hemicellulose isolated from sugar beet pulp.

Hemicelluloses Extraction Conditions	*M_w_*	*M_n_*	PD = *M_w_*/*M_n_*
Switchgrass Hemicellulose Acetylated, DS = 0.21			
5% NaOH, 50 °C, 3 h	23,500	5200	4.51
10% NaOH, 50 °C, 3 h	85,700	20,610	4.10
18% NaOH, 50 °C, 3 h	3500	460	7.59
Switchgrass Hemicellulose Acetylated, DS = 0.78			
10% NaOH, 50 °C, 3 h	85,200	20,900	4.05
Sugar Beet Pulp Hemicellulose			
10% KOH, 15 °C, 16 h	91,330	6920	13.05
24% KOH, 15 °C, 2 h	21,990	6320	3.48
8% NaOH, 15 °C, 16 h	88,850	10,650	8.34
18% NaOH, 15 °C, 2 h	21,620	6490	3.33

**Table 3:** The molecular weight of hemicellulose extracted from grasses and pinewood (10% NaOH) measured for acetylated samples with GPC (THF as the eluent).

Biomass	M_w_ (g.mol^−1^)	M_n_ (g.mol^−1^)	PD = M_w_/M_n_
Pine Wood	79,800	20,050	3.9
Switchgrass	85,710	20,670	4.2
Coastal Bermuda Grass	83,200	20,830	4

**Table 4:** Effect of reaction on the properties of foam based on switchgrass hemicellulose-DTPA/chitosan.

HC-DTPA-CS Reacting Time (h) T = 110 °C	Apparent Density	Void Fraction	Weight Loss (%), 1 h		Absorption (g/g)	
			Water	NaCl (0.3%)	Water	NaCl (0.3%)
0.1	1.093	0.993	32 ± 4	27 ± 2	9 ± 1	10 ± 3
1	1.093	0.994	30 ± 3	27 ± 1	14 ± 2	10 ± 1
1.5	1.095	0.996	24 ± 1	15 ± 3	17 ± 2	19 ± 1
2	1.097	0.997	13 ± 1	−10 ± 2	22 ± 1	25 ± 3
2.5	1.098	0.999	8 ± 2	−24 ± 4	29 ± 3	34 ± 5

**Table 5:** Properties of hemicellulose gels and the resulting coatings. HST stands for Hercules Size Test, with larger numbers indicating more water resistance. (*) Lower viscosity is attributed to agglomeration and poor dispersion of the gel. (n/d) not determined.

	Uncoated Sheet	0% AZC	5% AZC	10% AZC	20% AZC
Viscosity (MPa·s)	n/d	n/d	1000	1270	620 *
Gloss (%)	9.0	21.1	30.6	15.2	n/d
PPS roughness (mm)	18.0	8.6	7.5	11.0	n/d
HST (seconds)	1950	2260	2680	2810	n/d

**Table 6:** This table encapsulates key comparisons, drawing from recent studies that underscore the complementary nature of hemicellulose and lignin in pushing biobased adhesives toward industrial viability.

Aspect	Hemicellulose-Based Adhesives	Lignin-Based Adhesives	Hybrid Systems
Chemical Structure	Branched polysaccharide (e.g., xylans, mannans)	Aromatic phenolic polymer	Combined polysaccharide-aromatic matrix
Key Advantages	Biodegradable, low-cost tunable viscosity	Hydrophobic, strong wet strength, natural binder	Enhanced strength, water resistance
Challenges	Poor water resistance without modifications	High viscosity, needs demethylation	Complex processing
Applications	Wood composites, hydrogels, and food packaging	Wood panels, high humidity bonds	Plywood, particleboards, and advanced composites
Recent Advances (2024–2025)	Molecular weight optimization for bonding; hydrogel composites	UF adhesive improvements; uncondensed lignins	Valorization of bioresidues for sustainable formulations

**Table 7:** Quantitative Performance Comparison of Hemicellulose Gels vs. Conventional Materials.

Application	Property	Hemicellulose Biogels	Conventional/Competing Materials
Water Treatment	Pb^2+^ Adsorption Capacity	2.9 mg/g (at pH 5)	Activated Carbon: 1–200 mg/g (varies significantly), with commercial versions often in the 40–50 mg/g range [54].
Adhesive/Coatings	Adhesive Strength	Up to 3.46 MPa	Urea-Formaldehyde (UF) resins: Often >1.0 MPa (Dry Strength) [55]
	Liquid Resistance (HST)	2810 s	Starch-based adhesives: Often require significant chemical modification to achieve water resistance [56].

## Data Availability

All data in this text are referred to in the references and articles.
